# Chaotic genetic structure and past demographic expansion of the invasive gastropod *Tritia neritea* in its native range, the Mediterranean Sea

**DOI:** 10.1038/s41598-020-77742-3

**Published:** 2020-12-10

**Authors:** Emilie Boissin, Valentina Neglia, Sandra Baksay, Dragos Micu, Levent Bat, Bulent Topaloglu, Valentina Todorova, Marina Panayotova, Claudia Kruschel, Nataliya Milchakova, Emanuela Voutsinas, Sajmir Beqiraj, Ina Nasto, Giorgio Aglieri, Marco Taviani, Lorenzo Zane, Serge Planes

**Affiliations:** 1 PSL Research University: EPHE-UPVD-CNRS, USR 3278 CRIOBE, Laboratoire d’Excellence « CORAIL », Université de Perpignan, 52 Avenue Paul Alduy, 66860 Perpignan Cedex, France; 2 Laboratoire Evolution & Diversite Biologique, University TOULOUSE III - Paul Sabatier, 118 route de Narbonne, 31062 Toulouse Cedex 09, France; 3 Romanian Waters National Authority, 127 Mircea cel Batran Blvd., 900592 Constanţa, Romania; 4 Department of Hydrobiology, Sinop University Faculty of Fisheries, 57000 Sinop, Turkey; 5 Faculty of Aquatic Sciences, Istanbul University, Ordu St No: 8, 34134 Istanbul, Turkey; 6 Institute of Oceanology-BAS (IO-BAS), P.O. Box 152, 9000 Varna, Bulgaria; 7 University of Zadar, Ul. Mihovila Pavlinovića, 23000 Zadar, Croatia; 8 Institute of Biology of the Southern Seas, 2 Nakhimov Ave., Sevastopol, Russia 299011; 9 Institute of Oceanography, Hellenic Centre for Marine Research, P.O. Box 712, 19013 Anavyssos, Greece; 10 Faculty of Natural Sciences, Department of Biology, University of Tirana, Bulevardi “Zogu I Parë”, 25/1, 1001 Tiranë, Albania; 11 Department of Biology, Faculty of Technical Sciences, Vlora University, 9401 Vlora, Albania; 12 Department of Earth and Marine Sciences (DiSTeM), University of Palermo, via Archirafi 28, 90123 Palermo, Italy; 13 Consorzio Nazionale Interuniversitario per le Scienze del Mare (CoNISMa), Piazzale Flaminio 9, 00196 Rome, Italy; 14 Institute of Marine Sciences (ISMAR), CNR, via Gobetti 101, 40129 Bologna, Italy; 15 Stazione Zoologica Anton Dohrn, Villa Comunale, 80121 Napoli, Italy; 16 Department of Biology, Woods Hole Oceanographic Institution, 266 Woods Hole Road, Woods Hole, MA 02543 USA; 17 Dipartimento di Biologia, Università di Padova, via U. Bassi/58B, 35121 Padua, Italy

**Keywords:** Ecology, Evolution

## Abstract

To better predict population evolution of invasive species in introduced areas it is critical to identify and understand the mechanisms driving genetic diversity and structure in their native range. Here, we combined analyses of the mitochondrial COI gene and 11 microsatellite markers to investigate both past demographic history and contemporaneous genetic structure in the native area of the gastropod *Tritia neritea*, using Bayesian skyline plots (BSP), multivariate analyses and Bayesian clustering. The BSP framework revealed population expansions, dated after the last glacial maximum. The haplotype network revealed a strong geographic clustering. Multivariate analyses and Bayesian clustering highlighted the strong genetic structure at all scales, between the Black Sea and the Adriatic Sea, but also within basins. Within basins, a random pattern of genetic patchiness was observed, suggesting a superimposition of processes involving natural biological effects (no larval phase and thus limited larval dispersal) and putative anthropogenic transport of specimens. Contrary to the introduced area, no isolation-by-distance patterns were recovered in the Mediterranean or the Black Seas, highlighting different mechanisms at play on both native and introduced areas, triggering unknown consequences for species’ evolutionary trajectories. These results of *Tritia neritea* populations on its native range highlight a mixture of ancient and recent processes, with the effects of paleoclimates and life history traits likely tangled with the effects of human-mediated dispersal.

## Introduction

The description of mechanisms driving genetic diversity and structure of invasive species in their native range has proven to be of considerable use to predict further evolution in their introduced areas^1–4^. However, it is always difficult to disentangle the natural effects of life history traits and historical events (such as past climate changes) over species geographic ranges from recent human-related range expansion^5^. This is even more true for species whose introduced area is close to the native range, and only genetic tools can discriminate between a natural spread or a human-mediated introduction^6–9^. Life history traits are considered to be major drivers of population genetic diversity and structure^10^. In particular, species with direct development (no planktonic stage) have reduced potential for dispersal and usually show strong genetic structure^11–13^. The effects of past climate changes on population genetic structure and distribution have also been extensively reported^14,15^. For instance, sea-level changes in the Pleistocene often led to population fragmentations thus creating a dynamic of expansion/contraction of populations over time^16^. In the last decades, cases of human-related range expansion have increasingly been reported and the natural patterns of biodiversity have been altered by artificial translocation patterns^17–19^. The superimposition of these various evolutionary processes makes it difficult to evaluate the potential outcome of the future of introduced species.

*Tritia neritea* (Linnaeus, 1758), a scavenging nassariid gastropod, is a direct developer, with juveniles hatching as crawl-aways from single-embryo capsules attached to hard substrates^20,21^. This species was long known in the literature as *Cyclope neritea*, but the former genus *Cyclope* was recently moved to *Tritia* based on genetic results^22^. Two living species are currently recognized in the study region^23–25^, i.e. *T. neritea* (Linnaeus, 1758) and *T. pellucida* (Risso, 1826). *Tritia neritea* is euryhaline, found in lagoonal and river-influenced sandy and muddy bottoms^26^, which results in discontinuities of populations, with high densities in lagoons, bays, estuaries^27–30^. Its native range spreads across the Mediterranean, Black and Azov Seas to the adjacent eastern Atlantic coasts of Morocco and south Iberian Peninsula^24,31–35^. Although presenting a patchy distribution as well^36^, the case of the Black Sea is somewhat different given that its average salinity ranges between 18 and 22 psu, suitable to euryhaline species such as *T. neritea*.

The evolutionary history of the species and genus is complex. According to Gili & Martinell^37^, *Tritia neritea* originates from an early Pliocene ancestor identified as *Cyclope migliorinii* (Bevilacqua, 1928). Because of the basin-wide geographic range of *T. migliorinii*, it is impossible to formulate a robust hypothesis about the center of origin of *T. neritea*. *Tritia migliorinii* was characterized by a planktotrophic development and went extinct in the Pleistocene whereas *T. neritea,* lacking a larval phase, survived^37^. Such Pliocene-planktotrophic/Recent-non-planktotrophic pairs are common among gastropods of the Mediterranean while the reason for such trend is not yet clearly understood^38^. Climate changes strongly influenced the evolutionary and biogeographic patterns of marine organisms, with sea-level fluctuations triggering isolation of populations. In particular, for the Mediterranean Sea, Plio-Pleistocene sea-level changes often led to population fragmentations shaping species distributions and population genetic structures of many fish or marine invertebrates^39–42^. However, in the specific case of euryhaline gastropods, such as *T. neritea*, this process is not so obvious. On one hand, the populations restricted to lagoons are, in theory, exposed to habitat loss during sea-level changes, thus compromising population survival. In contrast, populations that inhabit coastal nearshore bottoms may prove to be more resilient to disconnection of their habitat during sea-level fluctuations.

The natural history background of *Tritia neritea* clearly depicts a rather complex situation with respect to population connectivity through space and time. Furthermore, starting from the 1970s, *T. neritea* was recorded further North, in Galicia^43^, in the Bay of Biscay^34,44,45^ and up to the English Channel^46^. Mitochondrial sequence data demonstrated the introduction in these areas^45–47^ (as opposed to a natural spread of the species). However, the history of introduction seems to be complex, with multiple independent introduction events from several sources. For instance, the North Adriatic region was proposed as a source for the North Western Iberian Peninsula invasion with the Manila clam, *Ruditapes philippinarum*, trade as the likely vector^47^, whereas the North Western Mediterranean and Portuguese areas were pointed as sources for the Bay of Biscay’s invasive population with the oyster trade as vector^45^. These and other studies on *T. neritea* focused on the introduced areas and included only one to few localities within the native range. Only Simon-Bouhet et al.^46^ included multiple localities in the native range, however, they only analyzed mitochondrial markers. While microsatellite markers were developed^48^, they have never been used to further our collective understanding of the population genetic structure of *T. neritea*. To add further complexity to such an intricate distribution, some populations are thought to represent recent introduction events within the ‘native’ range, such is likely the case for the Gulf of Gabès^49^. Consequently, it may prove very challenging to track unequivocally clear dispersal pathways (natural and/or artificial) for this species, and a better understanding of the population structure in the native area is critical.

**Figure 1:**
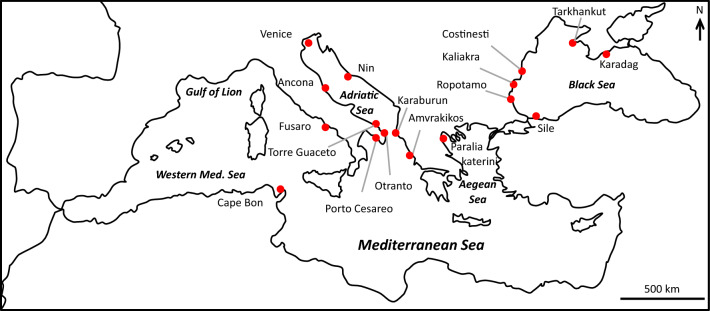
Map of the 17 localities sampled for *Tritia neritea* in the Mediterranean Sea.

Herein, we investigate the genetic structure and demographic history of *Tritia neritea* within its native range throughout the Mediterranean and the Black Seas (Fig. 1; Table 1). We aim to decipher biogeographic patterns considering the geological and climatic history of the region, with multiple disconnections of the Black Sea and the Adriatic Sea from the main Mediterranean region during low sea levels and subsequent recolonization following sea level rises^50–53^. In particular, population genetic structures of species from the Black Sea have seldom been assessed, as well as their relation to other populations in the Mediterranean Sea. However, a recent study on the black scorpionfish *Scorpaena porcus* clearly showed restricted gene flow between these two basins and demographic expansions of populations dated after the last glacial maximum. Therefore, the combination of the past history of the Mediterranean Sea, the patchy habitat distribution of the species and the low potential for dispersal resulting from the reproductive mode are likely to have shaped the evolutionary history of *T. neritea* and resulted in strong population structures at all scales. To test this, our study combines analyses of the mitochondrial COI gene and 11 microsatellite markers to investigate both past demographic history and contemporary genetic structure within the native geographic distribution of *T. neritea*, using coalescent theory and Bayesian skyline reconstruction, multivariate analyses and Bayesian clustering.

**Table 1:** Samples of *Tritia neritea* analyzed in this study and summary statistics.

Sea	Country	Locality	Code	Latitude	Longitude	N	A	A_T_	A_P_	Ho	He	F_IS_
**Adriatic**	Greece	Amvrakikos	AMV	39.040833	20.767778	47	11.54	127	6	0.527	0.682	0.229***
	Albania	Karaburun	KAP	40.515277	19.416944	45	8	88	8	0.449	0.587	0.238***
	Croatia	Nin	KOR-A	44.243888	15.179444	38	7.73	85	2	0.522	0.676	0.230***
	Italy	Venice	VEN	45.335398	12.345750	47	9.27	102	1	0.504	0.681	0.266***
	Italy	Ancona	ANC	43.706822	13.239722	10	5.91	65	–	0.489	0.724	0.339***
	Italy	Torre Guaceto	TOG	40.716650	17.800050	15	5.09	56	–	0.522	0.612	0.151**
	Italy	Otranto	OTR	40.203889	18.454444	45	8.34	92	2	0.531	0.673	0.214***
	Italy	Porto Cesareo	POC	40.242222	17.908333	44	8.45	93	3	0.552	0.655	0.159***
		TOTAL				291	18.64	205	23	0.520	0.737	0.295***
**Black**	Ukraine	Karadag	KAR	45.075833	35.413056	48	7.91	87	6	0.540	0.655	0.100***
	Ukraine	Tarkhankut	TAR	45.335556	32.969444	48	6.64	73	2	0.403	0.518	0.224***
	Romania	Costinesti	COS A	43.779392	28.582447	24	5.18	57	1	0.356	0.486	0.272***
	Romania	Costinesti	COS B	43.779392	28.582447	24	5.36	59	–	0.439	0.564	0.227***
	Romania	Costinesti	COS2	43.779392	28.582447	43	7.18	79	2	0.412	0.568	0.278***
	Bulgaria	Kaliakra	KAL1	43.361308	28.083861	47	7.55	83	2	0.404	0.602	0.331***
	Bulgaria	Kaliakra	KAL2	43.381806	28.470725	48	7	77	–	0.427	0.567	0.250***
	Bulgaria	Ropotamo Kitten	ROK	42.328717	27.752161	48	8.18	90	7	0.424	0.566	0.261***
	Turkey	Sile	SIL	41.175683	29.599567	46	8.64	95	1	0.517	0.643	0.197***
		TOTAL				376	17.00	189	22	0.450	0.752	0.379***
**W. Med**	Italy	Fusaro Lake	FUL	40.822257	14.050793	33	6.64	73	1	0.530	0.636	0.170***
	Tunisia	Tunis	TUN	36.789444	10.236222	41	6	66	–	0.452	0.515	0.123***
		TOTAL				74	9.18	101	3	0.487	0.618	0.213***
**Aegean**	Greece	Paralia Katerini	PAK	40.272653	22.600614	47	11.55	127	14	0.567	0.724	0.218***

N = number of specimens analyzed; A = mean number of alleles per sample; A_T_ = total number of alleles per sample; A_P_ = number of private alleles per sample; He = non-biased expected heterozygosity; F_IS_ = values of the inbreeding coefficient. Significance of F_IS_ values are given as follows: ***< 0.001; **< 0.01.

## Results

### Mitochondrial data

**Figure 2:**
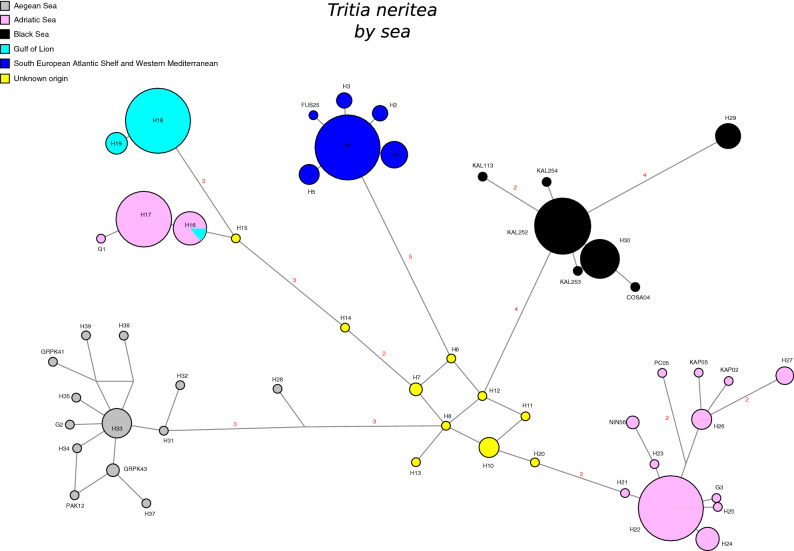
Median joining network of haplotypes of the 128 COI sequences of *Tritia neritea* generated for this study together with haplotypes available in GenBank. Circle sizes are proportional to the number of sequences per haplotype. Distances are proportional to the number of mutations between haplotypes. Numbers in red indicate numbers of mutations between haplotypes. The haplotypes marked as unknown origin were sampled in an introduced area, the Bay of Biscay^46^, but the population of origin in the native area is unknown.

A 524 bp portion of the COI gene was sequenced for 128 specimens, resulting in 20 haplotypes; 12 of which are new (Table 2). A strong geographic clustering is noticeable on the haplotype network colored by basins with 5 haplogroups visible and a central group (Fig. 2). Interestingly, the central group of haplotypes was only recorded in the invaded area and their source is unknown^51^. Samples from the Aegean Sea, the Black Sea and the South European Atlantic Shelf / Western Mediterranean Sea each form a distinct cluster of haplotypes. Samples from the Adriatic Sea are spread in the 2 remaining haplogroups, one with only Adriatic samples and the other one with samples from the Gulf of Lion (Western Mediterranean Sea). Noticeably, like most of the sequences from the west Mediterranean Sea already published (263 specimens), our 23 sequences from Fusaro lake and Cape Bon (Tunis) correspond to haplotype H1.

**Table 2:** Summary statistics of the Cytochrome oxidase I (COI) sequences of *Tritia neritea* analyzed in this study.

COI	N	H	Hd (SD)	Π (SD)	F	R_2_
Adriatic	27	8	0.678 (0.092)	0.0083 (0.0018)	0.735 ns	0.132 ns
Aegean	13	4	0.423 (0.164)	0.0014 (0.0006)	− 1.561 ns	0.193 ns
Black Sea	65	6	0.492 (0.053)	0.0011 (0.0002)	− 3.159*	0.112 ns
West Med	24	2	0.083 (0.075)	0.0002 (0.0001)	− 1.704 ns	0.167 ns

N = number of sequences; H = number of haplotypes; Hd (SD) = Haplotype diversity with standard deviation; Π (SD) = Nucleotide diversity with standard deviation; F = Fu & Li^115^ neutrality index; R_2_ = Ramos-Onsins & Rozas^116^ neutrality index.

**Figure 3:**
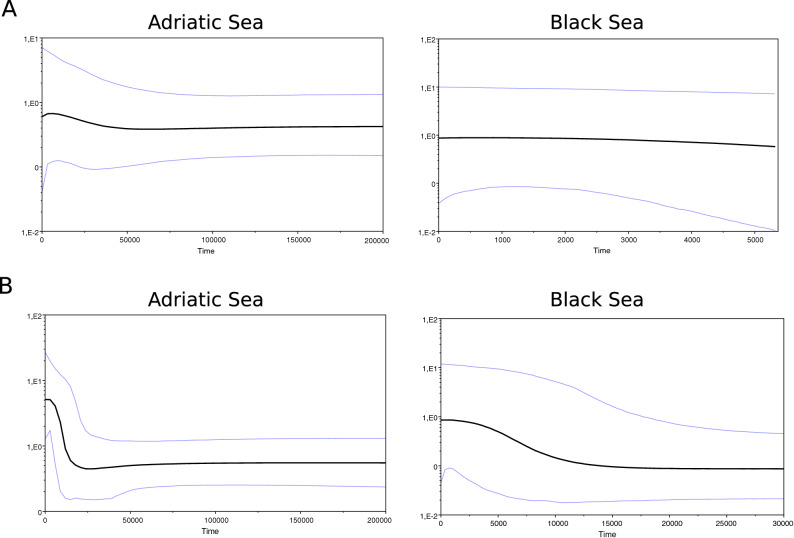
Bayesian Skyline Plots of *Tritia neritea* specimens from the Adriatic Sea and the Black Sea reconstructed from (**A**) sequences from this study only and (**B**) together with sequences already published. The X-axis indicates the time in years; the Y-axis indicates the female effective population size (NeT, with T = generation time). The black line is the median estimate of the estimated effective population size. The two blue lines are the upper and the lower estimates of 95% interval.

The Bayesian skyline plot did not show a population expansion based on only our samples from the Black Sea. However, a population expansion was detected for samples from the Adriatic Sea (Fig. 3A). When considering additional sequences available on GenBank, an expansion of population can also be distinguished for the Black Sea (Fig. 3B) and the signal of expansion in the Adriatic Sea becomes stronger (steeper slope in Fig. 3B). According to the molecular clock used, the age of expansion is datable at around 10,000–15,000 years ago, i.e. after the Last Glacial Maximum, for the Adriatic Sea population and around 5000–10,000 years ago for the Black Sea population.

### Microsatellite data

A total of 786 specimens were analyzed at 11 loci. Heterozygosity values (Ho) ranged from 0.356 to 0.567 and mean allele numbers ranged from 5.09 to 11.55 (Table 1). Fis values were high and significant for all populations sampled, ranging from 12 to 33% (Table 1), and this signal was not due to a single or few loci (data not shown). All pairwise Fst values were significantly different from zero, except those comparing Ancona and Venice in the northern Adriatic Sea, and Costinesti2 and CostinestiB in the Black Sea (Table 3).

**Table 3:** Pairwise F_st_ comparison of the 20 *Tritia neritea* samples.

FST	KAP	KOR-A	OTR	POC	ANC	TOG	VEN	AMV	COS2	ROK	KAL1	KAL2	COSA	COSB	SIL	KAR	TAR	PAK	FUL
KOR-A	0.180***																		
OTR	0.158***	0.091***																	
POC	0.132***	0.163***	0.189***																
ANC	0.154***	0.062***	0.033*	0.150***															
TOG	0.167***	0.206***	0.178***	0.130***	0.142***														
VEN	0.159***	0.088***	0.026**	0.183***	0.029ns	0.181***													
AMV	0.091***	0.108***	0.082***	0.125***	0.061***	0.166***	0.066***												
COS2	0.363***	0.292***	0.304***	0.270***	0.269***	0.338***	0.295***	0.301***											
ROK	0.349***	0.294***	0.298***	0.298***	0.300***	0.343***	0.288***	0.310***	0.295***										
KAL1	0.357***	0.294***	0.299***	0.271***	0.265***	0.316***	0.293***	0.299***	0.048***	0.265***									
KAL2	0.378***	0.301***	0.312***	0.280***	0.268***	0.347***	0.303***	0.307***	0.042***	0.313***	0.066***								
COSA	0.355***	0.301***	0.323***	0.310***	0.301***	0.368***	0.314***	0.309***	0.187***	0.241***	0.199***	0.261***							
COSB	0.354***	0.285***	0.297***	0.273***	0.266***	0.339***	0.291***	0.294***	0.002ns	0.282***	0.048***	0.067***	0.168***						
SIL	0.279***	0.197***	0.212***	0.244***	0.189***	0.285***	0.221***	0.234***	0.233***	0.187***	0.213***	0.252***	0.126***	0.205***					
KAR	0.299***	0.255***	0.260***	0.271***	0.245***	0.307***	0.245***	0.259***	0.218***	0.303***	0.183***	0.190***	0.193***	0.184***	0.124***				
TAR	0.416***	0.333***	0.353***	0.315***	0.332***	0.391***	0.345***	0.345***	0.223***	0.388***	0.235***	0.174***	0.409***	0.244***	0.360***	0.263***			
PAK	0.183***	0.111***	0.126***	0.176***	0.088***	0.210***	0.114***	0.119***	0.285***	0.264***	0.278***	0.295***	0.269***	0.277***	0.199***	0.232***	0.339***		
FUL	0.160***	0.132***	0.093***	0.191***	0.099***	0.196***	0.096***	0.082***	0.330***	0.341***	0.323***	0.335***	0.352***	0.324***	0.256***	0.276***	0.356***	0.178***	
TUN	0.293***	0.196***	0.193***	0.311***	0.226***	0.341***	0.182***	0.197***	0.418***	0.406***	0.406***	0.420***	0.427***	0.419***	0.332***	0.339***	0.441***	0.215***	0.144***

Significance of values are given as follows: ns = non-significant, ***< 0.001; **< 0.01; *< 0.05.

#### Among basins genetic differentiation

**Figure 4:**
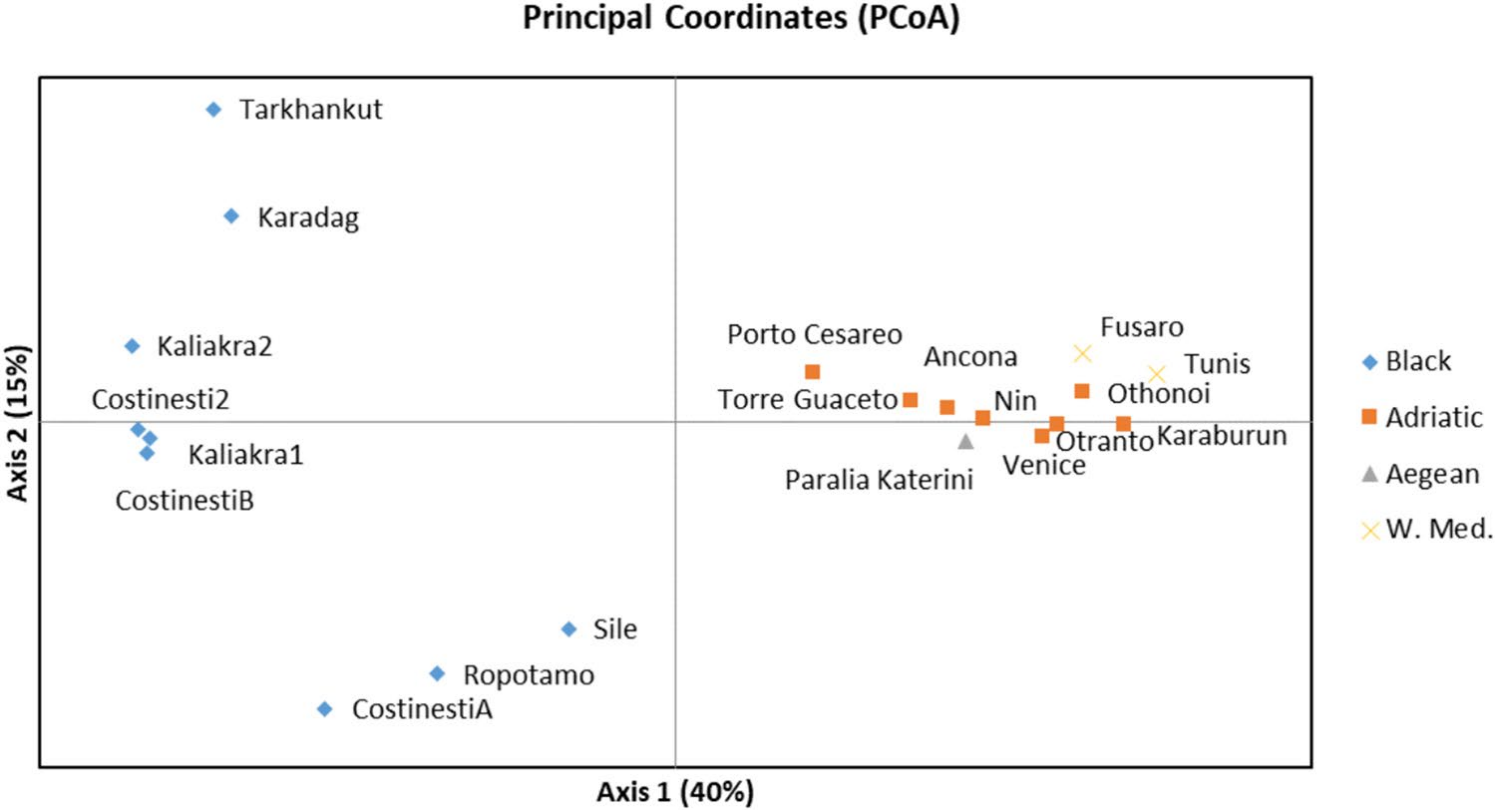
Principal Coordinates Analysis (PCoA) of the 17 localities sampled for *Tritia neritea* genotyped at 11 microsatellite markers. Coordinate 1 explains 40% of the variation while coordinate 2 explains 15% of the variation.

**Figure 5:**
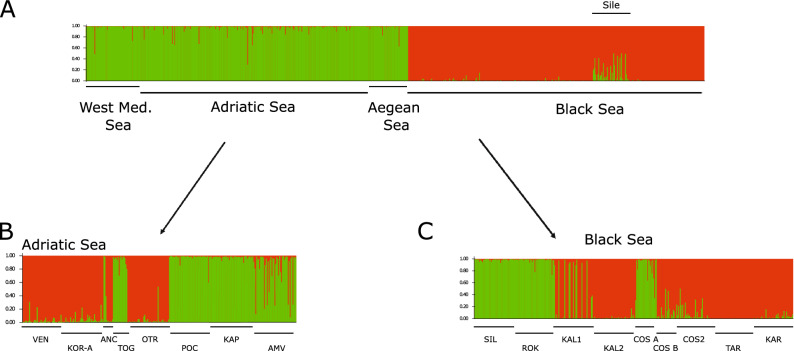
Bayesian clustering analysis showing the most likely partition for: (**A**) the total 786 multi-locus genotypes of *Tritia neritea* analyzed in this study; (**B**) the Adriatic samples only; (**C**) the Black Sea samples only, without Ropotamo.

The PCoA analysis showed the grouping of samples from the Western Mediterranean (Fusaro and Tunis) with samples from the Aegean Sea (Paralia Katerini) and samples from the Adriatic Sea (Fig. 4). Samples from the Black Sea clustered together. The Structure analysis on the total dataset revealed K = 2 groups as the most likely partitioning (Fig. S1). Using the sampling localities as prior (ocean basin) did not change the results (data not shown). Noticeably, the Western Mediterranean and the Aegean Sea samples clustered with samples from the Adriatic Sea and samples from the Black Sea form another cluster (Fig. 5). The sample from Sile shows a mixed genetic make-up, with some individuals presenting a proportion of their genome with an Adriatic Sea affinity.

#### Within basins genetic differentiation

For *T. neritea* in the Adriatic Sea, all the Fst values were significant, except for the Venice-Ancona pairwise comparison. The Structure plot revealed the most likely partitioning to be of K = 2 groups (Fig. 5B) corresponding to a first group composed of the northern localities (Ancona + Venice + Nin) together with Otranto in the south; and a second group with the southern localities (Karaburun, Porto Cesareo, Torre Guaceto, Amvrakikos). This grouping was also distinguishable on the PCoA (Supplementary Fig. S2). For *T. neritea* within the Black Sea, all the Fst values were significant. The Structure plot revealed K = 2 clusters to be most likely. The groups were (i) Costinesti2, Kaliakra1, Kaliakra2, CostinestiB; (ii) CostinestiA, Sile and Karadag, Tarkhankut; such as also separated on the PCoA (Fig. 4, Supplementary Fig. S1). Furthermore, the Mantel tests did not reveal any pattern of isolation by distance for both the Adriatic Sea (Z statistic = 6774, Pearson’s r = 0.09, *p* value > 0.05) and the Black Sea (Z statistic = 8629, Pearson’s r = 0.315, *p* value > 0.05) basins.

#### Parentage analysis

A total of 82 fullsib and 206 halfsib relationships were recovered among the 786 multilocus genotypes (Table S2). Most of the relationships occur within localities or close-by localities.

## Discussion

As expected for species characterized by a direct cycle without a pelagic larval stage, we observed genetically divergent populations even at local scales within small seas such as the Adriatic Sea or the Black Sea. The close investigation of this species within its native range demonstrates complex diversity patterns, genetic structure and historical changes that improve our understanding of patterns in the current and future introduced areas.

The mitochondrial dataset confirms a strong geographic clustering of haplotypes (Fig. 2), as already noted in^46,54,55^. Five mitochondrial clades correspond to marine ecoregions as defined in^56^: (i) South European Atlantic shelf and Western Mediterranean, (ii) Adriatic Sea, (iii) Aegean Sea and (iv) the Black Sea and (v) the 5th one is composed of specimens from both the Adriatic Sea and the Western Mediterranean Sea (Gulf of Lion). Noticeably, a group of haplotypes lies in the middle of the network, but was only recorded in the invaded area (North-Eastern Atlantic) while their source in the Mediterranean Sea remains unknown^54^. This group likely comes from an unsampled area of the native range (such as the South Eastern Mediterranean and Far Eastern Mediterranean basins) and its central position points toward an ancestral nature. As previously mentioned, if *T. neritea* originates from the widespread *T. migliorinii,* it is difficult to formulate a robust hypothesis about its center of origin. However, Gili & Martinell^37^ postulated a derivation of ‘*Cyclope*’ from a *Nassarius* (*Plicarcularia*) group containing two extant Mediterranean species (*Tritia gibbosula* and *T. circumcincta*), with a Levantine/southern Mediterranean distribution. Additionally, based on an east–west genetic diversity gradient, Simon-Bouhet^46^ hypothesized an eastern origin for *T. neritea*. Accordingly, our sample from the Aegean Sea demonstrates a high number of alleles and a high number of private alleles at nuclear loci. Further sampling on the South Eastern and Far Eastern Mediterranean coasts is warranted to locate and clarify the origin of these so far unsampled native haplotypes.

Whatever the origin of the species, the five mitochondrial lineages corresponding to distinct marine basins were likely differentiated during paleoclimate sea-level fluctuations. Simon-Bouhet^46^ hypothesized the persistence of *T. neritea* populations in several distinct refugia during glacial periods. Several refugia have been well documented in the Atlanto-Mediterranean basin^41,42,57^ corresponding to the Iberian Peninsula, the Western Mediterranean, the Eastern Mediterranean, etc. Based upon its current geographic distribution, *T. neritea* appears to be a temperate marine taxon. It might have responded to the lowered sea-surface temperature at glacial times, which favored the basinal spread of boreo-celtic species^58^, by shrinking its geographic range in the Mediterranean Sea to milder sectors of the basin^46^, e.g. the Levantine region. It is thus conceivable that we had geographically-distant stocks at the peak of the last glacial period in a disconnected mosaic-like situation. The post-glacial warming may have allowed for the progressive recolonization of sectors of the basin, resulting in the present pan-Mediterranean distribution. The high private allele number in Amvrakikos and Karaburun supports this hypothesis. Specimens from a refugium located in the Ionian Sea might have recolonized the Adriatic Sea, as seen in the decapod *Carcinus aestuarii*^59^. Likewise, the high private allele number at the Aegean location suggests a refugial area.

Therefore, our dataset seems consistent with the hypothesis that past climate changes impacted the genetic structure of *T. neritea*. Furthermore, the Bayesian skyline plots revealed population expansions in both seas when enough sequences are available (Fig. 3). Interestingly, the signal of population expansion is detectable even in the face of the strong genetic structure of *T. neritea*, a feature known to potentially hinder the detection of demographic expansion^60^. This expansion is dated from just after the Last Glacial Maximum for the Adriatic Sea population, as also seen for other marine invertebrates and fish in the region^53,61^ including other nassariid gastropod species^62^. In the specific case of the Black Sea, this basin achieved its current marine status only *ca.* 7000 years ago^63^. Before its post-glacial inundation, it was ascertained that the Black Sea, together with the Marmara Sea, was a lacustrine basin (Neoeuxinian stage) completely secluded from the Mediterranean Sea^63,64^. This fact rules out any chance that any marine refuges existed (even if euryhaline) within the Black Sea during the last glaciation, a theoretical claim put forth for other organisms but not applicable to *T. neritea*. In agreement with this age, fossil evidence from the Kerch Strait supports the occurrence of *T. neritea* in the Late Holocene^65^, thus setting a tempo for the Black Sea population presence in the basin. The settling of the modern Black Sea by *T. neritea* is thus a geologically-young phenomenon (< 7,000 years ago) that took place from the Aegean Sea via the Marmara Sea. Interestingly, the expansion of population for the Black Sea is dated at 5000–10,000 ya, which would agree with the timeframe of the recolonization of this sea proposed in the literature.

Regarding genetic diversity, overall, the heterozygosity at nuclear loci of *Tritia neritea* in its native range (0.486 < He < 0.724, Table 1) is comparable to that of other gastropods^66–68^, although lower than the values for another direct developing gastropod species *Crepidula convexa*^52^ (0.885 < He < 0.943) or the broadcast spawner *C. fornicata*^69^ (0.806 < He < 0.838). Additionally, all the F_is_ values are high and significant, demonstrating a heterozygote deficiency in every populations. Such high values are not surprising for a species with a lack of planktonic larval phase, thus prone to inbreeding^50^ but were also detected for the mussel *Mytilus galloprovincialis,* a species with long-lived pelagic larvae^70^. Heterozygote deficiency is a common phenomenon in marine molluscs^71^. Some of the proposed explanations to this phenomenon are null alleles, local inbreeding, selection against heterozygotes and life history traits that produce spatial or temporal population substructure leading to a Wahlund effects^70–73^. Parentage analysis (Table S2) revealed half-sib (206 pairs) and full-sib (82 pairs) relationships within localities or between very close localities, and family structure could thus explain some of the heterozygote deficiencies detected. With a low dispersal potential for *T. neritea*, a sample likely comprises multiple related individuals, but also multiple families, which would trigger a high genetic diversity together with significant F_is_ values. Such Wahlund effect on multiple families with different allelic frequencies has been suggested for the *Siphonaria* limpets^72^. Other direct developer gastropods such as *Crepidula convexa* in the USA also display high and significant values of F_is_^51,52^. Other direct developer marine invertebrates in the Mediterranean Sea also show high levels of inbreeding^12,74,75^.

Regarding genetic differentiation between basins, the pairwise F_st_ values, PCoA and Bayesian clustering all showed genetic differentiation between the Black Sea and the Mediterranean Sea populations, with the Aegean Sea and the Western Mediterranean populations clustering with the Adriatic population. There are also more private alleles in the Adriatic Sea than in the Black Sea. Genetic diversity and total number of alleles are also lower in the Black Sea compared to the Adriatic Sea (in spite of a higher number of specimens collected in the Black Sea), as previously noticed for several species of fish^53,76,77^. Only few studies have investigated population genetics for species distributed both in the Black Sea and the rest of the Mediterranean Sea, however a genetic differentiation appears to be the trend for the fish populations studied so far^53,78–81^. Furthermore, the mussel *Mytilus galloprovincialis* also showed strong genetic differentiation between its populations of the Black Sea and the rest of the Mediterranean Sea^70^. Some explanations for these differentiations can be found in the oceanographic currents, the presence of narrow straits (e.g. Bosphorus) and specific environmental conditions (in particular, low salinity) of the Black Sea^80,81^. Additionally, the Otranto Strait and the Aegean Front have been demonstrated to influence population genetic structures, however to a lesser extent for species without long pelagic larval phases compared to species with long pelagic larval phases^82^. Noticeably, the relatedness of individuals within basins could also trigger some proportion of the differentiation detected, as family groups are known to influence the inferences made with the program Structure^83,84^. However, recent literature advises against purging related individuals in population genetics^85^, since the removal of individuals also reduces precision of genetic estimates. This holds particularly true when errors in pedigree reconstructions are expected, as in our case due to the limited number of loci. Finally, discrepancies between the Atlantic and the Mediterranean population structures also exist. In the Mediterranean, we did not find a signal of isolation-by-distance, contrary to what was shown in the Atlantic population of *T. neritea* using the COI mitochondrial gene^46,47,55^ . This discrepancy might be linked to the very distinct mechanisms at play during introductions on new areas and natural evolutionary history on the native areas. The introduced area was likely home to a linear spread along the Atlantic shore over a short period of time, with founding events typical of colonization of new areas^47^, whereas in the native area, the strong genetic structure and chaotic pattern are likely due to rare long-distance dispersal events in the Mediterranean Sea over evolutionary periods and relicts of glacial disconnected refuges.

Within basins, strong genetic structures were also detectable, however they were “unpatterned” or “random” with no clear geographic clustering. Importantly, the half-sib and full-sib relationships detected within and among localities might increase the number of detected clusters^83^, suggesting that these results should be taken with caution. The Adriatic Sea did not show an east/west genetic differentiation, contrary to a recent study on the scorpionfish, *Scorpaena porcus,* sampled at the same localities^53^. Here, a north/south differentiation seems to arise, however, not perfect, as the Otranto sample clusters with the northern localities and the Amvrakikos sample comprises individuals with genetic make-up of the northern cluster. Within the Black Sea, spatially close samples such as Costinesti A and B or Kaliakra 1 and 2 also show genetic differentiation. Life history traits, such as local vs. broadcast dispersal, are known to have strong effects on genetic structure^10,11^ and the strong genetic differentiation at local scales is thus not surprising for *T. neritea*, a species without a dispersal stage. The Polyplacophora *Lepidopleurus cajetanus*, with a low potential for dispersal (lecithotrophic larvae) also exhibited a ‘chaotic patchiness’ pattern in the Mediterranean basin defined by a high genetic variability with locality-exclusive haplotypes, high genetic divergence, and a lack of geographic structure^86^. The term “chaotic genetic patchiness” was defined for species with a potential for dispersal and that show strong genetic structure at scales smaller than their dispersal potential and where “instantaneous drift” between cohorts is common^87^. *Tritia neritea* has no larval dispersal phase but seems to be especially prone to demonstrate such “random” genetic patchiness. The mechanisms underlying this pattern remain unknown but are likely a mixture of ‘natural’ and ‘artificial’ processes. For direct developers, rafting on floating objects has been proposed as a surrogate^88,89^, and would likely be sporadic, not recurrent, and not linked to geography, thus responsible for creating this kind of genetic patchiness. Oyster culture is also a vector for the introduction of *Tritia neritea* on the Atlantic coast^45^ and populations could be moved similarly within the native range (see below).

Samples from Sile demonstrated a mixed genetic make-up at the nuclear loci compared to the remaining of the Black Sea populations. The mitochondrial sequences, however, belong to the Black Sea haplogroup. Similarly, Simon-Bouhet^54^, based on mito-nuclear discordance raised the possibility of the introduced nature of a population at Mar Menor (Western Mediterranean Sea) and Aissaoui et al.^49^ documented a recent introduction event in the Gulf of Gabès (Tunisia), both populations are located in the native area. Likewise, the mixed genetic make-up of our sample from Sile, might be due to recent introductions of Mediterranean specimens into the Black Sea. Sile has a marina for yachts and small to medium sized fishing boats, which make seasonal trips to the Marmara and Aegean Seas. The discrepancy between nuclear and mitochondrial markers could be explained by mitochondrial capture (i.e. complete introgression), a phenomenon known in many organisms^90–92^ and mitochondrial introgression has been reported for mollusks^93,94^. Alternatively, if only male specimens were introduced, the nuclear dataset would be of foreign origin while the mitochondrial marker would remain from the geographic location sampled. However, no data on sex ratio is available for *T. neritea* to support this second hypothesis. Further study is warranted to verify if this population shows any effects of admixture (i.e. mixing of specimens from different genetic clusters), such as hybrid vigor. Rius & Darling^95^ recently highlighted the unknown evolutionary trajectory for such admixed populations. Human activities are responsible for the translocation of vast amounts of organisms, altering natural patterns of dispersal and gene flow. Anthropogenic effects on the native area have been demonstrated in the ascidian *Ciona intestinalis* that displayed genetic homogeneity among both close and distant sites and dissimilar genetic composition between close sites^5^. Similarly, a recent study on hydroids revealed contrasted patterns of strong genetic structure on local and regional scales contrasting with some haplotypes shared among ocean basins, revealing the likely effect of human-mediated transport^96^.

## Conclusion

The patterns seen from our samples of *Tritia neritea* on its native range seem to be a mixture of recent and ancient processes, with the effects of paleoclimates and life history traits likely tangled with the effects of human-mediated dispersal. As anthropogenic pressure grows, it is going to be more and more difficult to disentangle the natural and artificial patterns of biodiversity.

## Methods

### Samples

In this study, through the application of the sampling scheme of the CoCoNet project^97^, a total of 786 specimens from 17 localities were analyzed (1 locality from the Aegean Sea, 2 localities from the West Mediterranean Sea, 8 localities from the Adriatic Sea + Ionian Sea (herein the 3 localities in the Ionian Sea close to the Adriatic Sea will be broadly included in the Adriatic Sea localities) and 6 localities from the Black Sea, see Fig. 1 and Table 1 for details). Samples were collected from the infralittoral by snorkeling or diving at a depth between 1 and 5 m, in 2013–2014. DNA was extracted from foot muscle using the PureGene protocol with a QIAxtractor robot (QiaGen, Hilden, Germany).

### Molecular analyses

#### Mitochondrial sequences

A portion of the Cytochrome oxidase subunit 1 (COI) was amplified for a subset of 128 specimens including all locations, using the specific primers Cy2 5′-GTTAAAATTTCGATCTGTTA-3′ and CyR 5′-GGATTAGTTGGTACAGC-3′^45^. PCR mixture and cycling parameters were as given in this publication. The 42 haplotypes available from GenBank^45–47,55^ together with their frequencies were added to this newly generated dataset in order to place our samples in a broader context. Notably, a group of these haplotypes lies in the middle of the haplotype network, but have been only recorded in the invaded area and their source remains unknown^54^.

#### Microsatellite markers

A set of 14 microsatellite markers were screened for this study, 7 markers are from Simon-Bouhet et al.^48^ and 7 come from a new microsatellite library (Genoscreen, Lille, France) generated specifically for this study (see Supplementary Table S1 for locus details). PCR were performed in 10 µl volume containing: 5 µL of QIAGEN Multiplex PCR master mix, 3 µL of RNase free water (provided with the QIAGEN type-it Multiplex PCR Master Mix), 0.02 µl of each primer (100 µM) and 1 µl of DNA template. Three multiplexes of four, five and five loci, respectively, were run for each specimen. Cycling parameters were as follows: 15 min at 95 °C followed by 30 cycles of: (i) 30 s at 94 °C, (ii) 1 min 30 s at the optimal annealing temperature (55 °C, 57 °C or 60 °C) and (iii) 1 min at 72 °C and a final step at 57 °C during 30 min. Amplifications were verified on 1% Agarose gel. PCR products were sent to a private company for genotyping (GenoScreen, Lille, France). Genotypes were scored using GeneMapper v4.0 (Applied Biosystems).

### Data analyses

#### Mitochondrial sequences

COI sequences were aligned using Mafft v.7 online^98^. Haplotype and nucleotide diversity indices as well as neutrality test statistics were computed in DnaSP v.5^99^. Haplotype networks were computed with Network v5.0.0.3 (www. fluxus.engineering.com) using the Median Joining algorithm^100^. Mr AIC^101^ was used to determine the best fit model of nucleotide substitutions. Changes in population sizes were investigated using the Bayesian skyline plot (BSP) framework in Beast 2^102^. As the assumptions of this analysis framework are that the sequences represent a small sample from a haploid population evolving under Wright–Fisher dynamics, the BSPs were computed for each basin separately (Adriatic Sea / Black Sea) in order to try and work on samples that are closer to a single panmictic population. A total of 10,000,000 generations sampled every 100th generation were run. As no mutation rate is calibrated for *Tritia neritea*, a mean mutation rate ranging from 0.012 to 0.016 (substitutions per site per My), used for the COI gene in Protostomia^103^ and in limpets^104^ was applied.

#### Microsatellite markers

The presence of null alleles, scoring errors or large allele drop-out were verified using micro-checker^105^. As null alleles are common in mollusks^106^, we decided to discard the three microsatellite markers that presented an estimated frequency of null alleles above 0.10. Further analyses were run on the dataset with 11 loci. Summary statistics were computed in Genetix v.4.05.02^107^: the mean number of alleles (A), the expected (He) and observed (Ho) heterozygosities, the inbreeding coefficient (F_is_) and the fixation index (F_st_). The correction for multiple comparisons of Benjamini & Hochberg^108^ was applied to the F_st_ *p* values. A Principal Coordinate Analysis (PCoA) was computed in Genalex v6.5^109^. The most likely number of clusters present in our dataset was estimated using Structure v2.3^110^. This analysis was run both with and without prior on the location. After several first runs, the parameters were set as follows: a burn-in period of 100,000 iterations followed by 500,000 recorded iterations for K = 1 to K = 8 clusters and 15 iterations per K values. The most probable number of clusters present in this dataset was determined using the Evanno’s *Δ*K approach^111^ using Structure Harvester online^112^. Finally, a pattern of Isolation-by-distance was tested by a Mantel test in Genetix using the genetic distance [F_st_/(1 − F_st_)]^113^ and the log coastal geographical distance (in km) between each pair of localities within basins. Significance was obtained using a random permutation procedure implemented in Genetix (5,000 permutations). Finally, a parentage analysis was run using the software Colony^114^. Even if the number of loci is low for this kind of analyses^84^, and given that heterozygote deficiency is often detected in mollusk population genetic analyses^71^, we wanted to verify any sign of family structure in our dataset. This relatedness analysis was performed on the whole dataset including the 786 multilocus genotypes and with the following input parameters: a mating system with female and male polygamy, with possible inbreeding and without clones. The method was composed of 3 runs set as ‘long’, with a full likelihood method and a likelihood precision that was set to ‘high’.

## Supplementary information

Supplementary Table 1.

Supplementary Table 2.

Supplementary Figure 1.

Supplementary Figure 2.

## Data Availability

Sequences are available in GenBank (accession numbers: MN577950–MN577969).
