# Supplementary material for: Chaotic genetic structure and past demographic expansion of the invasive gastropod Tritia neritea in its native range, the Mediterranean Sea
Source: Sci Rep. 2020 Dec 10;10:21624. doi: 10.1038/s41598-020-77742-3 (PMC7730386; doi:10.1038/s41598-020-77742-3)
Supplement: Supplementary file 1 — Supplementary Table 1. [file 41598_2020_77742_MOESM1_ESM.docx]

Chaotic genetic structure and past demographic expansion of the invasive gastropod *Tritia neritea* in its native range, the Mediterranean Sea.

Boissin E^1*^, Neglia V^1^, Baksay S^1,2^, Micu D^3^, Bat L^4^, Topaloglu B^5^, Todorova V^6^, Panayotova M^6^, Kruschel C^7^, Milchakova N^8^, Voutsinas E^9^, Beqiraj S^10^, Nasto I^11^, Aglieri G^12,13^, Taviani M^14,15,16^, Zane L^13,17^, Planes S^1^

**Supplementary Table S1** Microsatellite name, repeat, motif, and sequences of primer F and R

| **Locus name** | **Repeat** | **Motif** | **Primer F** | **Primer R** |
| --- | --- | --- | --- | --- |
| cons 805 | di | gt | AGAACGACGTCTCGAGCATT | TAAGGCCAAGGACAGGAGAA |
| cons 463 | tri | cat | CACGACTACTGCCACACCAC | GGTGTTGGTGATTATGTTGGC |
| concat2 | tri | gat | CACCCCGTTTAGAGCATTAG | GATTACCATCACCAGGGTCG |
| DEFSP | di | ac | CAGTCGCAAAACTTGGCATA | CATTTGTTGCATCATTTCCG |
| DJ8FX | tetra | gttt | TTTGTTGGGTTAGTTAGTGAGTGAGTT | GCTGAAGTTGGTCTTTAAGAAGC |
| C5AWQ | tri | atc | CCAACAGCAATGTCATCATAGG | TGATGATTGTGATGATAGCGG |
| DPNMY | tetra | gaca | CGTCAGACAGATAGACATACAGGG | GTCCTTCTGCCTGCCTCTTT |
