# Supplementary material for: Chaotic genetic structure and past demographic expansion of the invasive gastropod Tritia neritea in its native range, the Mediterranean Sea
Source: Sci Rep. 2020 Dec 10;10:21624. doi: 10.1038/s41598-020-77742-3 (PMC7730386; doi:10.1038/s41598-020-77742-3)
Supplement: Supplementary file 2 — Supplementary Table 2. [file 41598_2020_77742_MOESM2_ESM.docx]

| Chaotic genetic structure and past demographic expansion of the invasive gastropod *Tritia neritea* in its native range, the Mediterranean Sea. |
| --- |
| Boissin E^1*^, Neglia V^1^, Baksay S^1,2^, Micu D^3^, Bat L^4^, Topaloglu B^5^, Todorova V^6^, Panayotova M^6^, Kruschel C^7^, Milchakova N^8^, Voutsinas E^9^, Beqiraj S^10^, Nasto I^11^, Aglieri G^12,13^, Taviani M^14,15,16^, Zane L^13,17^, Planes S^1^ |
|  |
| Table S2. Fullsib and Halfsib relationships recovered from the parentage analysis using the software Colony (Jones et al. 2010). |

| **Fullsibs** | **offspring 1** | **offspring 2** | **Probability** |
| --- | --- | --- | --- |
|  | AL_KAP29 | AL_KAP41 | 1.000 |
|  | BU_ROK27 | BU_ROK41 | 1.000 |
|  | BU_KAL1_54 | BU_KAL1_65 | 1.000 |
|  | BU_KAL2_4 | BU_KAL2_7 | 1.000 |
|  | RO_COSA_10 | RO_COSA_24 | 1.000 |
|  | TU_SIL22 | TU_SIL32 | 1.000 |
|  | UK_TAR05 | UK_TAR41 | 1.000 |
|  | UK_TAR15 | UK_TAR28 | 1.000 |
|  | TU_TUN_21 | TU_TUN_38 | 1.000 |
|  | GR_AMV_37 | GR_AMV_38 | 0.999 |
|  | RO_COS2_12 | RO_COS2_32 | 0.999 |
|  | RO_COS2_45 | RO_COSB_01 | 0.999 |
|  | BU_ROK11 | BU_ROK43 | 0.999 |
|  | RO_COSA_09 | RO_COSA_10 | 0.999 |
|  | UK_TAR09 | UK_TAR12 | 0.999 |
|  | TU_TUN_05 | TU_TUN_18 | 0.999 |
|  | TU_TUN_18 | TU_TUN_23 | 0.999 |
|  | BU_KAL1_58 | BU_KAL2_38 | 0.998 |
|  | BU_KAL2_14 | BU_KAL2_31 | 0.998 |
|  | TU_SIL03 | TU_SIL43 | 0.998 |
|  | UK_TAR30 | UK_TAR36 | 0.998 |
|  | TU_TUN_09 | TU_TUN_39 | 0.998 |
|  | AL_KAP01 | AL_KAP25 | 0.997 |
|  | IT_TG02 | IT_TG08 | 0.997 |
|  | CR_NIN_07 | CR_NIN_34 | 0.996 |
|  | BU_ROK07 | BU_ROK14 | 0.996 |
|  | IT_ANC07 | IT_ANC08 | 0.995 |
|  | IT_TG09 | IT_TG12 | 0.995 |
|  | BU_KAL2_31 | BU_KAL2_5 | 0.995 |
|  | TU_SIL06 | TU_SIL27 | 0.995 |
|  | IT_PC09 | IT_PC29 | 0.994 |
|  | BU_ROK18 | BU_ROK21 | 0.994 |
|  | BU_KAL2_10 | BU_KAL2_34 | 0.994 |
|  | UK_TAR33 | UK_TAR46 | 0.994 |
|  | IT_PC33 | IT_PC41 | 0.993 |
|  | BU_ROK31 | BU_ROK33 | 0.993 |
|  | BU_KAL1_55 | RO_COSA_10 | 0.993 |
|  | UK_TAR11 | UK_TAR13 | 0.992 |
|  | TU_TUN_30 | TU_TUN_44 | 0.992 |
|  | RO_COS2_14 | RO_COS2_44 | 0.991 |
|  | BU_KAL1_55 | RO_COSA_24 | 0.991 |
|  | UK_TAR25 | UK_TAR48 | 0.991 |
|  | RO_COS2_26 | RO_COSA_19 | 0.990 |
|  | BU_KAL2_1 | BU_KAL2_26 | 0.990 |
|  | BU_KAL1_55 | RO_COSA_09 | 0.989 |
|  | BU_KAL1_72 | BU_KAL1_89 | 0.989 |
|  | RO_COSA_16 | RO_COSA_23 | 0.989 |
|  | UK_TAR17 | UK_TAR18 | 0.989 |
|  | IT_ANC12 | IT_ANC13 | 0.988 |
|  | BU_ROK22 | BU_ROK42 | 0.988 |
|  | RO_COS2_01 | RO_COS2_45 | 0.987 |
|  | RO_COSA_15 | RO_COSA_16 | 0.987 |
|  | TU_TUN_24 | TU_TUN_40 | 0.987 |
|  | UK_KAR31 | UK_KAR48 | 0.986 |
|  | IT_TG05 | IT_TG13 | 0.983 |
|  | BU_ROK17 | BU_ROK32 | 0.982 |
|  | UK_KAR37 | UK_KAR38 | 0.982 |
|  | IT_VEN31 | IT_VEN44 | 0.981 |
|  | RO_COSA_15 | RO_COSA_17 | 0.981 |
|  | UK_TAR06 | UK_TAR09 | 0.981 |
|  | BU_ROK21 | BU_ROK29 | 0.978 |
|  | AL_KAP19 | AL_KAP34 | 0.974 |
|  | RO_COS2_25 | RO_COS2_34 | 0.974 |
|  | IT_FUL34 | IT_FUL38 | 0.972 |
|  | BU_KAL1_68 | BU_KAL1_76 | 0.971 |
|  | TU_TUN_34 | TU_TUN_43 | 0.971 |
|  | TU_SIL24 | TU_SIL37 | 0.969 |
|  | TU_TUN_06 | TU_TUN_12 | 0.969 |
|  | UK_KAR04 | UK_KAR34 | 0.966 |
|  | AL_KAP14 | AL_KAP32 | 0.965 |
|  | BU_ROK03 | BU_ROK46 | 0.965 |
|  | UK_TAR40 | UK_TAR44 | 0.963 |
|  | RO_COS2_43 | BU_KAL1_77 | 0.962 |
|  | BU_KAL2_3 | BU_KAL2_8 | 0.961 |
|  | CR_NIN_37 | CR_NIN_39 | 0.957 |
|  | IT_PC07 | IT_PC31 | 0.954 |
|  | CR_NIN_02 | CR_NIN_45 | 0.951 |
|  | BU_ROK05 | BU_ROK30 | 0.951 |
|  | IT_PC08 | IT_PC33 | 0.950 |
|  | BU_KAL1_56 | BU_KAL1_78 | 0.950 |
|  | IT_FUL14 | IT_FUL33 | 0.950 |
|  | TU_TUN_09 | TU_TUN_20 | 0.950 |
| **Halfsibs** | **offspring 1** | **offspring 2** | **Probability** |
|  | IT_FUL08 | IT_FUL34 | 0.998 |
|  | TU_TUN_01 | TU_TUN_37 | 0.997 |
|  | GR_AMV_34 | GR_AMV_38 | 0.996 |
|  | UK_KAR14 | UK_KAR31 | 0.996 |
|  | GR_PAK01 | GR_PAK36 | 0.996 |
|  | IT_FUL27 | IT_FUL34 | 0.996 |
|  | IT_OTR10 | IT_OTR15 | 0.995 |
|  | IT_OTR10 | GR_PAK13 | 0.995 |
|  | GR_PAK06 | GR_PAK40 | 0.995 |
|  | UK_TAR19 | UK_TAR21 | 0.994 |
|  | GR_PAK39 | GR_PAK47 | 0.994 |
|  | TU_SIL26 | TU_SIL30 | 0.993 |
|  | GR_PAK29 | GR_PAK36 | 0.993 |
|  | IT_FUL21 | IT_FUL48 | 0.993 |
|  | TU_TUN_10 | TU_TUN_34 | 0.993 |
|  | TU_TUN_16 | TU_TUN_28 | 0.993 |
|  | BU_KAL1_69 | BU_KAL1_78 | 0.992 |
|  | GR_PAK23 | GR_PAK32 | 0.992 |
|  | IT_FUL27 | IT_FUL38 | 0.992 |
|  | CR_NIN_14 | CR_NIN_32 | 0.991 |
|  | RO_COSA_05 | RO_COSA_18 | 0.991 |
|  | GR_PAK34 | GR_PAK44 | 0.990 |
|  | AL_KAP29 | GR_AMV_24 | 0.989 |
|  | TU_SIL08 | TU_SIL29 | 0.989 |
|  | GR_PAK06 | GR_PAK15 | 0.989 |
|  | BU_KAL1_83 | RO_COSA_24 | 0.988 |
|  | BU_KAL2_11 | RO_COSB_15 | 0.988 |
|  | TU_SIL11 | TU_SIL13 | 0.988 |
|  | UK_KAR27 | UK_KAR36 | 0.988 |
|  | GR_PAK16 | GR_PAK32 | 0.988 |
|  | IT_FUL40 | IT_FUL43 | 0.987 |
|  | IT_PC33 | IT_PC34 | 0.987 |
|  | BU_ROK22 | BU_ROK43 | 0.987 |
|  | UK_KAR08 | UK_KAR15 | 0.987 |
|  | UK_KAR10 | UK_KAR38 | 0.987 |
|  | UK_KAR13 | UK_KAR24 | 0.987 |
|  | GR_PAK03 | GR_PAK06 | 0.987 |
|  | IT_FUL45 | IT_FUL48 | 0.987 |
|  | TU_TUN_11 | TU_TUN_28 | 0.987 |
|  | TU_TUN_12 | TU_TUN_39 | 0.987 |
|  | BU_ROK41 | BU_ROK43 | 0.986 |
|  | UK_TAR22 | UK_TAR46 | 0.986 |
|  | TU_TUN_24 | TU_TUN_25 | 0.986 |
|  | IT_OTR13 | GR_AMV_16 | 0.985 |
|  | RO_COS2_17 | BU_KAL1_96 | 0.985 |
|  | UK_KAR10 | UK_KAR22 | 0.985 |
|  | UK_KAR42 | UK_KAR44 | 0.985 |
|  | GR_PAK01 | GR_PAK40 | 0.985 |
|  | GR_PAK38 | GR_PAK43 | 0.985 |
|  | TU_TUN_03 | TU_TUN_14 | 0.985 |
|  | TU_TUN_13 | TU_TUN_28 | 0.985 |
|  | AL_KAP09 | GR_AMV_15 | 0.984 |
|  | CR_NIN_35 | CR_NIN_46 | 0.984 |
|  | RO_COS2_29 | BU_KAL1_82 | 0.984 |
|  | TU_TUN_16 | TU_TUN_29 | 0.984 |
|  | BU_ROK32 | BU_ROK34 | 0.983 |
|  | TU_SIL28 | TU_SIL44 | 0.983 |
|  | UK_KAR04 | UK_KAR15 | 0.983 |
|  | UK_TAR13 | UK_TAR20 | 0.983 |
|  | IT_PC08 | IT_PC34 | 0.982 |
|  | IT_PC08 | IT_PC35 | 0.982 |
|  | UK_KAR01 | UK_KAR14 | 0.982 |
|  | TU_SIL08 | TU_SIL41 | 0.981 |
|  | GR_PAK21 | GR_PAK37 | 0.981 |
|  | TU_SIL12 | TU_SIL40 | 0.980 |
|  | UK_KAR36 | UK_KAR40 | 0.980 |
|  | IT_FUL22 | TU_TUN_26 | 0.980 |
|  | GR_PAK22 | GR_PAK43 | 0.979 |
|  | TU_TUN_09 | TU_TUN_37 | 0.979 |
|  | AL_KAP21 | GR_AMV_11 | 0.978 |
|  | CR_NIN_48 | IT_VEN47 | 0.978 |
|  | IT_OTR47 | IT_VEN47 | 0.978 |
|  | RO_COSA_11 | TU_SIL06 | 0.978 |
|  | TU_SIL29 | TU_SIL35 | 0.978 |
|  | IT_OTR19 | IT_OTR31 | 0.977 |
|  | BU_ROK09 | BU_ROK21 | 0.977 |
|  | BU_KAL1_63 | BU_KAL2_7 | 0.977 |
|  | RO_COS2_37 | BU_KAL1_97 | 0.976 |
|  | UK_KAR26 | UK_KAR42 | 0.976 |
|  | UK_TAR09 | UK_TAR26 | 0.976 |
|  | UK_TAR27 | UK_TAR46 | 0.976 |
|  | TU_TUN_08 | TU_TUN_23 | 0.976 |
|  | AL_KAP03 | AL_KAP34 | 0.975 |
|  | RO_COS2_11 | BU_KAL1_64 | 0.975 |
|  | BU_ROK14 | BU_ROK19 | 0.975 |
|  | TU_SIL13 | TU_SIL21 | 0.975 |
|  | UK_KAR16 | UK_TAR42 | 0.975 |
|  | IT_FUL12 | IT_FUL25 | 0.975 |
|  | IT_FUL13 | IT_FUL18 | 0.974 |
|  | GR_AMV_30 | GR_AMV_44 | 0.974 |
|  | RO_COS2_29 | BU_KAL1_84 | 0.974 |
|  | BU_ROK24 | BU_ROK31 | 0.974 |
|  | BU_KAL1_58 | RO_COSB_08 | 0.974 |
|  | RO_COSA_05 | RO_COSA_11 | 0.974 |
|  | UK_KAR32 | UK_KAR41 | 0.974 |
|  | TU_TUN_04 | TU_TUN_37 | 0.974 |
|  | TU_TUN_14 | TU_TUN_38 | 0.974 |
|  | CR_NIN_23 | CR_NIN_48 | 0.973 |
|  | IT_OTR12 | IT_TG05 | 0.973 |
|  | IT_PC35 | IT_PC41 | 0.973 |
|  | BU_ROK04 | BU_ROK38 | 0.973 |
|  | UK_TAR02 | UK_TAR23 | 0.973 |
|  | UK_TAR15 | UK_TAR35 | 0.973 |
|  | BU_KAL1_82 | BU_KAL2_13 | 0.972 |
|  | GR_PAK20 | GR_PAK27 | 0.972 |
|  | CR_NIN_07 | CR_NIN_33 | 0.971 |
|  | CR_NIN_17 | IT_VEN23 | 0.971 |
|  | BU_ROK18 | BU_ROK36 | 0.971 |
|  | UK_KAR12 | UK_KAR40 | 0.971 |
|  | UK_TAR15 | UK_TAR19 | 0.971 |
|  | GR_PAK26 | GR_PAK31 | 0.971 |
|  | TU_SIL02 | TU_SIL31 | 0.970 |
|  | UK_KAR04 | UK_KAR41 | 0.970 |
|  | UK_TAR15 | UK_TAR16 | 0.970 |
|  | GR_PAK06 | GR_PAK38 | 0.970 |
|  | TU_TUN_37 | TU_TUN_44 | 0.970 |
|  | CR_NIN_25 | CR_NIN_44 | 0.969 |
|  | IT_VEN32 | IT_VEN34 | 0.969 |
|  | RO_COS2_37 | BU_KAL1_51 | 0.969 |
|  | TU_SIL14 | TU_SIL35 | 0.969 |
|  | TU_TUN_08 | TU_TUN_11 | 0.969 |
|  | IT_OTR09 | IT_OTR36 | 0.968 |
|  | IT_OTR46 | IT_VEN41 | 0.968 |
|  | IT_PC33 | IT_PC45 | 0.968 |
|  | IT_ANC04 | IT_VEN05 | 0.968 |
|  | IT_VEN41 | IT_VEN44 | 0.968 |
|  | IT_FUL19 | IT_FUL48 | 0.968 |
|  | IT_TG06 | IT_TG08 | 0.967 |
|  | UK_KAR27 | UK_KAR30 | 0.967 |
|  | GR_PAK13 | GR_PAK44 | 0.967 |
|  | IT_OTR30 | IT_OTR35 | 0.966 |
|  | BU_ROK07 | BU_ROK19 | 0.966 |
|  | UK_KAR01 | UK_KAR26 | 0.966 |
|  | UK_KAR14 | UK_KAR48 | 0.966 |
|  | IT_OTR04 | IT_ANC07 | 0.965 |
|  | BU_KAL1_58 | BU_KAL2_1 | 0.965 |
|  | BU_KAL2_12 | BU_KAL2_21 | 0.965 |
|  | TU_SIL06 | TU_SIL40 | 0.965 |
|  | UK_KAR03 | UK_KAR17 | 0.965 |
|  | AL_KAP23 | GR_AMV_11 | 0.964 |
|  | IT_ANC03 | IT_VEN06 | 0.964 |
|  | IT_VEN23 | IT_VEN39 | 0.964 |
|  | BU_KAL2_19 | UK_TAR44 | 0.964 |
|  | TU_TUN_03 | TU_TUN_08 | 0.964 |
|  | IT_FUL07 | IT_FUL09 | 0.963 |
|  | IT_OTR32 | IT_OTR40 | 0.963 |
|  | IT_PC08 | IT_PC40 | 0.963 |
|  | RO_COSA_01 | TU_SIL33 | 0.963 |
|  | GR_PAK04 | GR_PAK25 | 0.962 |
|  | GR_PAK39 | GR_PAK43 | 0.962 |
|  | IT_FUL21 | IT_FUL45 | 0.962 |
|  | RO_COS2_29 | BU_KAL1_80 | 0.961 |
|  | BU_ROK30 | BU_ROK32 | 0.961 |
|  | TU_SIL02 | TU_SIL42 | 0.961 |
|  | TU_SIL17 | TU_SIL40 | 0.961 |
|  | UK_KAR12 | UK_KAR36 | 0.961 |
|  | IT_OTR34 | IT_OTR48 | 0.960 |
|  | BU_KAL1_67 | TU_SIL09 | 0.960 |
|  | TU_SIL04 | TU_SIL11 | 0.960 |
|  | TU_TUN_11 | TU_TUN_29 | 0.960 |
|  | BU_ROK39 | BU_ROK43 | 0.959 |
|  | BU_KAL1_97 | BU_KAL2_29 | 0.959 |
|  | AL_KAP31 | GR_AMV_11 | 0.958 |
|  | AL_KAP40 | IT_OTR45 | 0.958 |
|  | IT_OTR28 | IT_VEN46 | 0.958 |
|  | IT_PC13 | IT_PC16 | 0.958 |
|  | IT_VEN13 | IT_VEN47 | 0.958 |
|  | IT_VEN27 | GR_AMV_29 | 0.958 |
|  | BU_KAL2_11 | BU_KAL2_12 | 0.958 |
|  | IT_FUL35 | TU_TUN_42 | 0.958 |
|  | BU_KAL1_85 | BU_KAL2_20 | 0.957 |
|  | BU_KAL1_98 | RO_COSA_14 | 0.957 |
|  | IT_FUL34 | IT_FUL45 | 0.957 |
|  | AL_KAP17 | AL_KAP18 | 0.956 |
|  | IT_OTR28 | IT_VEN27 | 0.956 |
|  | GR_PAK06 | GR_PAK20 | 0.956 |
|  | GR_PAK10 | GR_PAK15 | 0.956 |
|  | RO_COS2_01 | BU_KAL1_69 | 0.955 |
|  | RO_COS2_06 | RO_COSB_24 | 0.955 |
|  | RO_COS2_18 | RO_COS2_38 | 0.955 |
|  | BU_KAL1_69 | RO_COSB_24 | 0.955 |
|  | BU_KAL2_19 | UK_TAR47 | 0.955 |
|  | UK_KAR01 | UK_KAR44 | 0.955 |
|  | UK_TAR26 | UK_TAR28 | 0.955 |
|  | IT_PC33 | IT_PC35 | 0.954 |
|  | GR_AMV_01 | GR_AMV_22 | 0.954 |
|  | GR_AMV_27 | GR_AMV_30 | 0.954 |
|  | GR_AMV_31 | GR_AMV_46 | 0.954 |
|  | RO_COS2_44 | BU_KAL1_89 | 0.954 |
|  | BU_ROK17 | BU_ROK31 | 0.954 |
|  | BU_KAL1_53 | BU_KAL1_82 | 0.954 |
|  | AL_KAP39 | IT_PC11 | 0.953 |
|  | IT_OTR39 | IT_VEN11 | 0.953 |
|  | IT_VEN14 | IT_VEN16 | 0.953 |
|  | BU_KAL1_85 | BU_KAL2_30 | 0.953 |
|  | BU_ROK04 | BU_ROK36 | 0.952 |
|  | BU_KAL2_26 | BU_KAL2_38 | 0.952 |
|  | CR_NIN_12 | CR_NIN_15 | 0.951 |
|  | CR_NIN_35 | CR_NIN_38 | 0.951 |
|  | IT_TG13 | IT_TG14 | 0.951 |
|  | IT_FUL13 | IT_FUL27 | 0.951 |
|  | AL_KAP29 | AL_KAP33 | 0.950 |
|  | IT_PC02 | IT_PC30 | 0.950 |
|  | IT_PC04 | IT_PC11 | 0.950 |
|  | UK_TAR16 | UK_TAR38 | 0.950 |
|  | GR_PAK14 | GR_PAK23 | 0.950 |
