# Supplementary material for: Chaotic genetic structure and past demographic expansion of the invasive gastropod Tritia neritea in its native range, the Mediterranean Sea
Source: Sci Rep. 2020 Dec 10;10:21624. doi: 10.1038/s41598-020-77742-3 (PMC7730386; doi:10.1038/s41598-020-77742-3)

Chaotic genetic structure and past demographic expansion of the invasive gastropod *Tritia neritea* in its native range, the Mediterranean Sea.

Boissin E, Neglia V, Baksay S, Micu D, Bat L, Topaloglu B, Todorova V, Panayotova M, Kruschel C, Milchakova N, Voutsinas E, Beqiraj S, Nasto I, Aglieri G, Taviani M, Zane L, Planes S

Supplementary Figure S1. Results of the Structure runs: Likelihood value plots and Evanno's delta K distribution for the whole dataset and for each basin separately

Total dataset

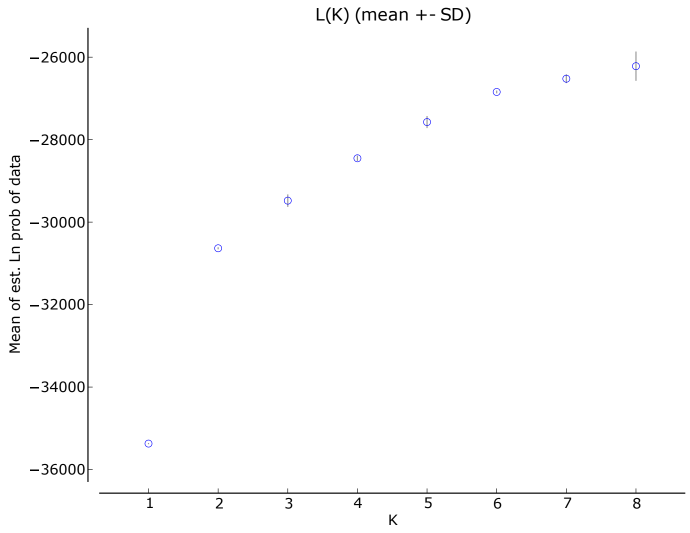

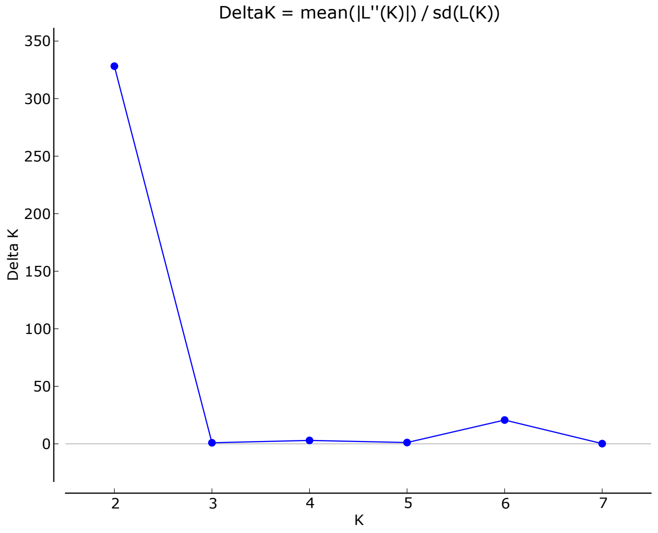

Adriatic only

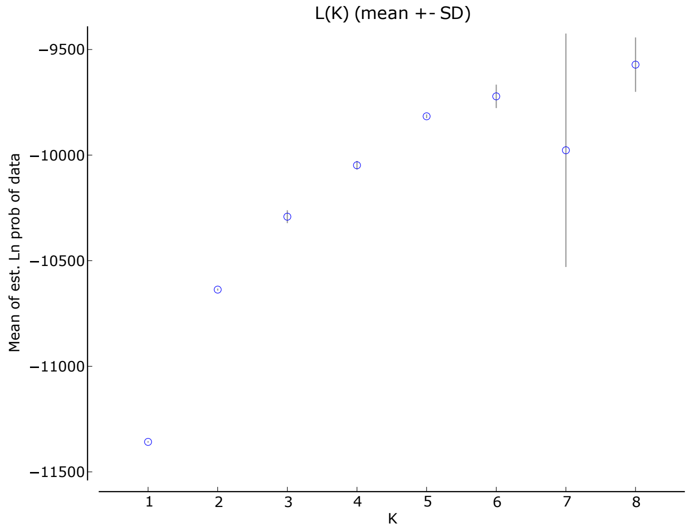

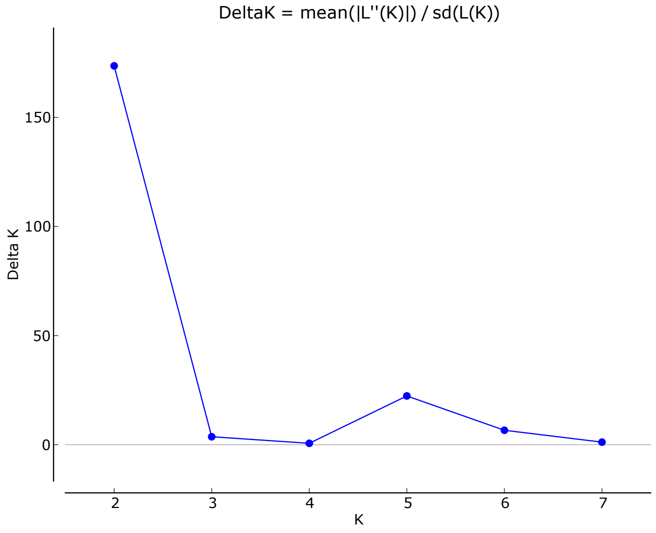

Black Sea only

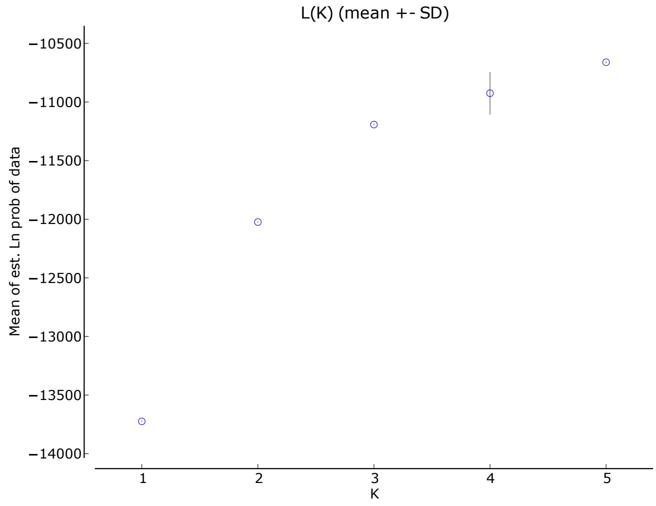

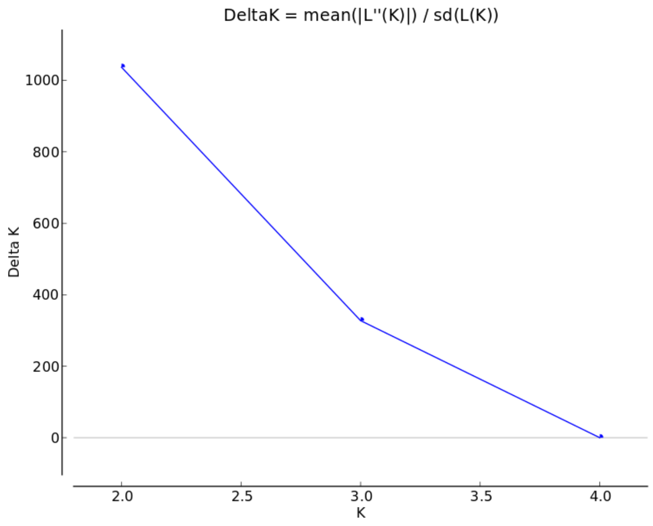

Supplement: Supplementary file 3 — Supplementary Figure 1. [file 41598_2020_77742_MOESM3_ESM.pdf]
