# Supplementary material for: Chaotic genetic structure and past demographic expansion of the invasive gastropod Tritia neritea in its native range, the Mediterranean Sea
Source: Sci Rep. 2020 Dec 10;10:21624. doi: 10.1038/s41598-020-77742-3 (PMC7730386; doi:10.1038/s41598-020-77742-3)

Chaotic genetic structure and past demographic expansion of the invasive gastropod *Tritia neritea* in its native range, the Mediterranean Sea.

Boissin E^1*^, Neglia V^1^, Baksay S^1,2^, Micu D^3^, Bat L^4^, Topaloglu B^5^, Todorova V^6^, Panayotova M^6^, Kruschel C^7^, Milchakova N^8^, Voutsinas E^9^, Beqiraj S^10^, Nasto I^11^, Aglieri G^12,13^, Taviani M^14,15,16^, Zane L^13,17^, Planes S^1^

**Supplementary Figure S2.** Principal Coordinates Analysis (PCoA) of *Tritia neritea* specimens separated by oceanographic basins: A) the Adriatic Sea and B) the Black Sea


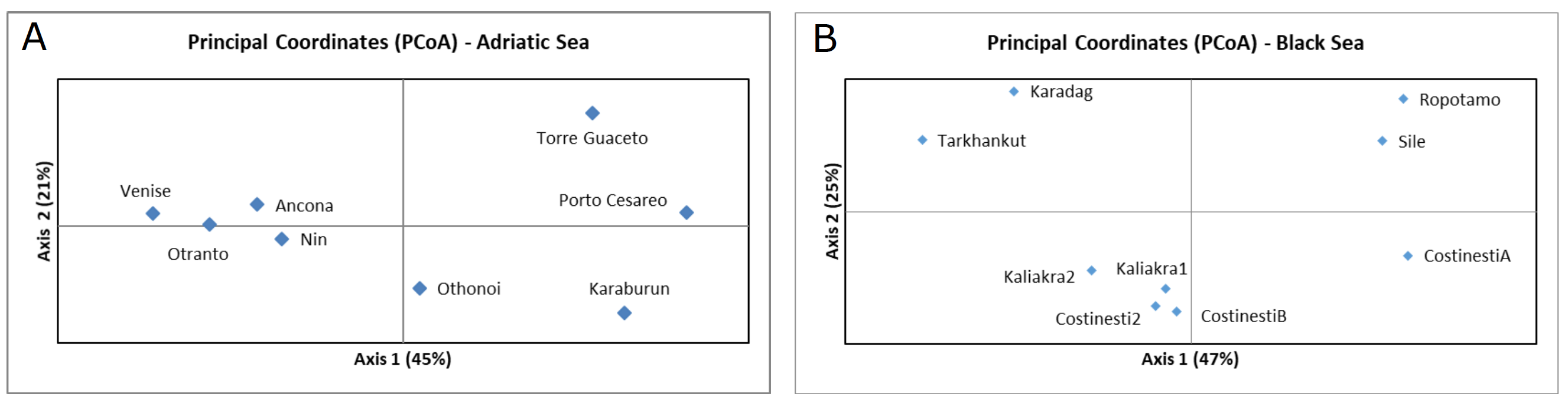

Supplement: Supplementary file 4 — Supplementary Figure 2. [file 41598_2020_77742_MOESM4_ESM.docx]
